# Functionalization of α-synuclein fibrils

**DOI:** 10.3762/bjnano.6.12

**Published:** 2015-01-12

**Authors:** Simona Povilonienė, Vida Časaitė, Virginijus Bukauskas, Arūnas Šetkus, Juozas Staniulis, Rolandas Meškys

**Affiliations:** 1 Department of Molecular Microbiology and Biotechnology, Institute of Biochemistry, Vilnius University, Mokslininku 12, Vilnius LT-08662, Lithuania; 2 Semiconductor Physics Institute, Center for Physical Sciences and Technology, A. Gostauto 11, Vilnius LT-01108, Lithuania; 3 Institute of Botany of Nature Research Center, Zaliuju Ezeru 49, LT-08406 Vilnius, Lithuania

**Keywords:** α-synuclein, atomic force microscopy, gold nanoparticles, nanostructures, self-assembly

## Abstract

The propensity of peptides and proteins to form self-assembled structures has very promising applications in the development of novel nanomaterials. Under certain conditions, amyloid protein α-synuclein forms well-ordered structures – fibrils, which have proven to be valuable building blocks for bionanotechnological approaches. Herein we demonstrate the functionalization of fibrils formed by a mutant α-synuclein that contains an additional cysteine residue. The fibrils have been biotinylated via thiol groups and subsequently joined with neutravidin-conjugated gold nanoparticles. Atomic force microscopy and transmission electron microscopy confirmed the expected structure – nanoladders. The ability of fibrils (and of the additional components) to assemble into such complex structures offers new opportunities for fabricating novel hybrid materials or devices.

## Introduction

Due to their ability to form self-assembled structures, amyloid proteins have become a very attractive material in the field of nanobiotechnology [1]. Many proteins or peptides can form amyloids under appropriate experimental conditions and recent studies suggest that amyloid formation is a generic property of the polypeptide chain [2]. Proteins harbouring β-sheet-rich domains are more prone to rapid fibril formation [3]. Many studies are directed towards the revelation of these amyloidogenic motif sequences to get more information about the formation of amyloids as this process is usually associated with the development of human neurodegenerative diseases and microbial physiological processes [4]. The principles of self-assembly of amyloidogenic elements together with their observed polymorphism have been found to be beneficial for the design and development of novel nanostructures and nanomaterials from the bottom up [5]. These natural building blocks with a wide range of modifiable properties have become very attractive tools for applications in biotechnology, material science, molecular electronics and related fields [6]. A variety of nanostructures, including nanotubes, nanospheres, nanofibers, nanotapes and hydrogels, have been investigated by a combination of techniques such as atomic force microscopy (AFM), transmission electron microscopy (TEM), measurement of thioflavin T (ThT) fluorescence, etc. [6–7].

In this study, α-synuclein (α-Syn), the amyloid protein that is linked to several neurodegenerative diseases including Parkinson’s disease [8–9], was chosen as a building block element. α-Syn is a small 140 amino acid long protein that is highly soluble and heat-stable. Three regions can be recognized in α-Syn: a highly conservative N-terminal region, a variable internal non-amyloid component (NAC) (which is responsible for the fibrillization), and an unfolded acidic C-terminal region [8,10] (which has chaperone-like activity) [11]. Purified α-Syn is a natively unfolded protein at neutral pH [12], but under appropriate conditions it can aberrantly polymerize into fibrils with typical amyloid properties [13]. The distinct features (e.g., size, shape, secondary structure) of in vitro-assembled α-Syn fibrils can be modulated by varying experimental conditions such as pH, ionic strength, temperature, etc. [14–16]. Also, several factors, including oxidative stress, post-translational modifications, proteolysis, and the concentration of fatty acids, phospholipids and metal ions, were shown to induce and/or modulate the α-Syn structure and oligomerization in vitro [17–18]. The formation of fibrils is enhanced under acidic conditions (pH 4–6) and the morphology of matured fibrils is influenced by the shape of any preformed aggregates [14,19] and mutations in the amino acid sequence of the protein [20].

From a medical point of view, the aggregation of proteins including α-Syn is unfavourable in vivo, yet, amyloid fibrils have a potential to be engineered into novel, proteinous, nanoscale materials and devices [21–22]. Amyloids, including α-Syn, show stability against harsh physical, chemical, and biochemical conditions. Such extraordinary properties make them attractive nanomaterials for a variety of applications [23–24], including the development of a scaffold for enzyme immobilization [25–26], for tissue engineering [27–28], as well as use as a template for fibril metallization [29–35] or for the biomineralization of fibrils [36]. Nanostructures are usually designed by modifying proteins or peptides prior to fibril assembly [21,37–41]. Although post-assembly functionalization remains a significant objective, the studies where fibrils have been modified after fibrillization have appeared in recent years. For example, a method to functionalize in vitro-grown, insulin, amyloid fibrils with various inorganic materials leading to the formation of apatite and platinum complex structures ordered by the amyloid template has been described [36]. Ries et al., recently developed a method for super resolution imaging of amyloid fibrils with binding-activated probes where unlabeled target structures (eg., α-synuclein fibrils) can be visualised after the amyloid-specific fluorophore binds to the target and becomes highly fluorescent [42]. Reches and Gazit also demonstrated the chemical and biological decoration of aromatic dipeptide nanotubes with biotin moiety and avidin [43]. Finally, the functionalization of amyloids with enzymes and other tags that are suitable for the desired application offers unique tools for the design and development of bionanosensing devices and other functional platforms [22,26]. Therefore, the functionalization of the amyloid fibrils is a promising approach for the creation of interfibrillar structures as well as for the development of the multicomponent biocomplexes that can be employed in a broad range of applications.

In this paper, we report the construction of a new nanostructure – nanoladders, which was generated from amyloid fibrils formed by the α-SynC141 mutant protein. Genetically inserted cysteine residue allowed the chemical modification of the target protein. Novel well-ordered multicomponent nanoderivatives have been constructed by labelling α-SynC141 fibrils with biotin. The biotinylated fibrils have been further assembled with neutravidin-conjugated gold nanoparticles. The resulting structures were evaluated by AFM and TEM. Amyloid-based hybrid nanostructures described in this paper reveal new insights into the general principles underlying the regularities of multistage self-assembly process and can be exploited in the construction of nanobiomaterials.

## Results

### Construction and purification of α-SynC141

**Figure 1:**
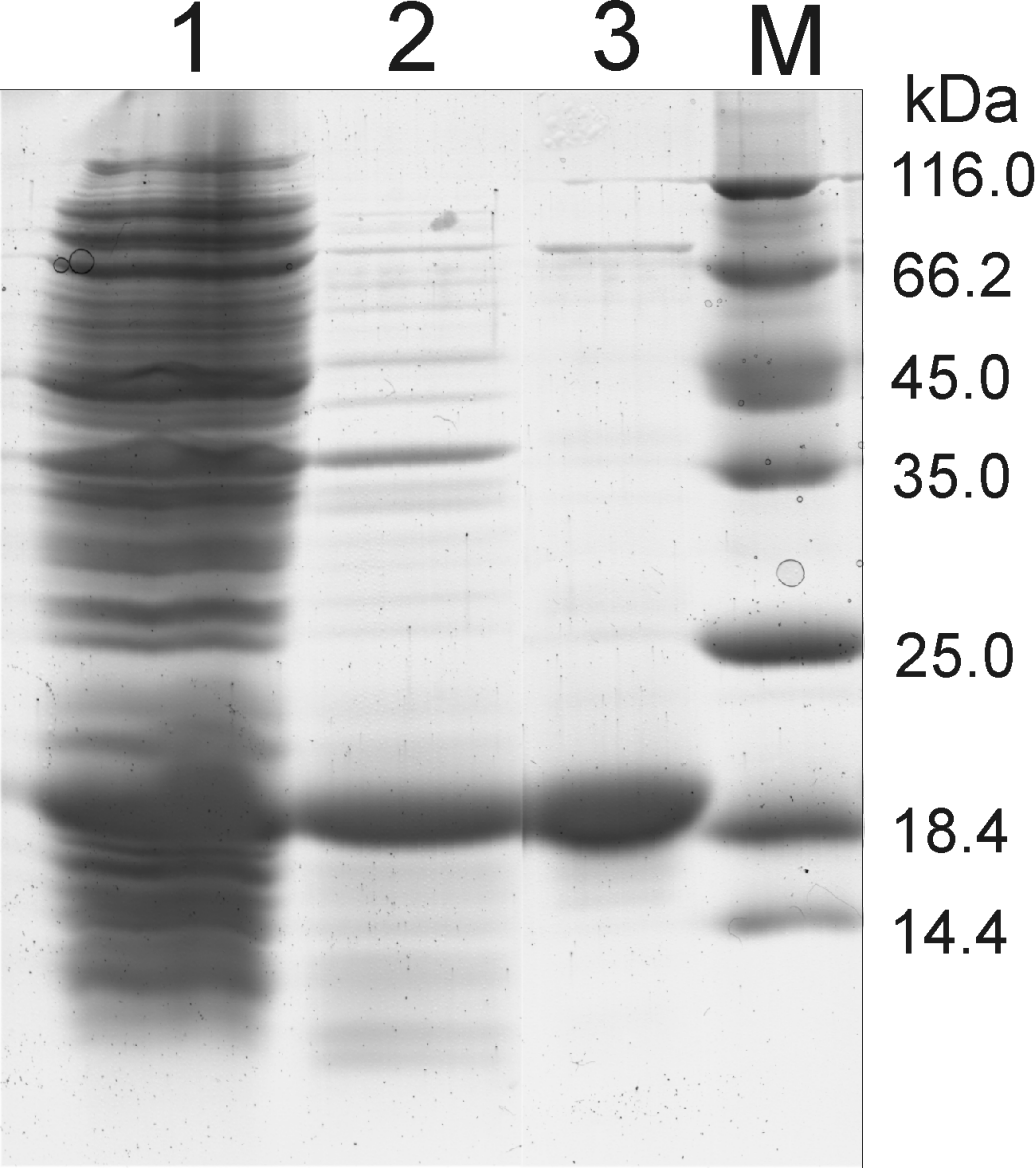
SDS-PAGE profile of α-SynC141 at different steps during purification. 1: cell-free extract; 2: cell-free extract after heating at 100 °C; 3: α-SynC141 protein after purification using Q XL and ANX columns; M: a molecular mass marker (unstained protein molecular weight marker (Thermo Scientific)). The positions of the molecular weight standards are indicated to the right (kDa).

The mutant α-SynC141 was constructed as described in the Experimental section. After expression in *E. coli* BL21 (DE3) cells, α-SynC141 was found as a soluble protein and the level of expression was the same as that of a native α-Syn. The presence of the additional amino acid in the C-terminus did not affect the solubility of the α-SynC141 protein, and it remained soluble even after being subjected to heating. However, after incubation at 100 °C and centrifugation, the vast majority of cell protein was removed, and additional purification steps were necessary. First, the recombinant protein was purified with a HiTrap ANX column and reloaded into a Q XL column. A typical yield from 1 L of culture was 30 mg of the homogenous protein. A band corresponding to an 18 kDa protein was observed in 15% sodium dodecyl sulfate polyacrylamide gel electrophoresis (SDS-PAGE) (Figure 1).

The theoretical molecular mass of α-SynC141 is 14.562 kDa. The presence of the introduced cysteine residue was confirmed by mass spectroscopy analysis (data not shown).

### Investigation of self-assembly of α-SynC141

**Figure 2:**
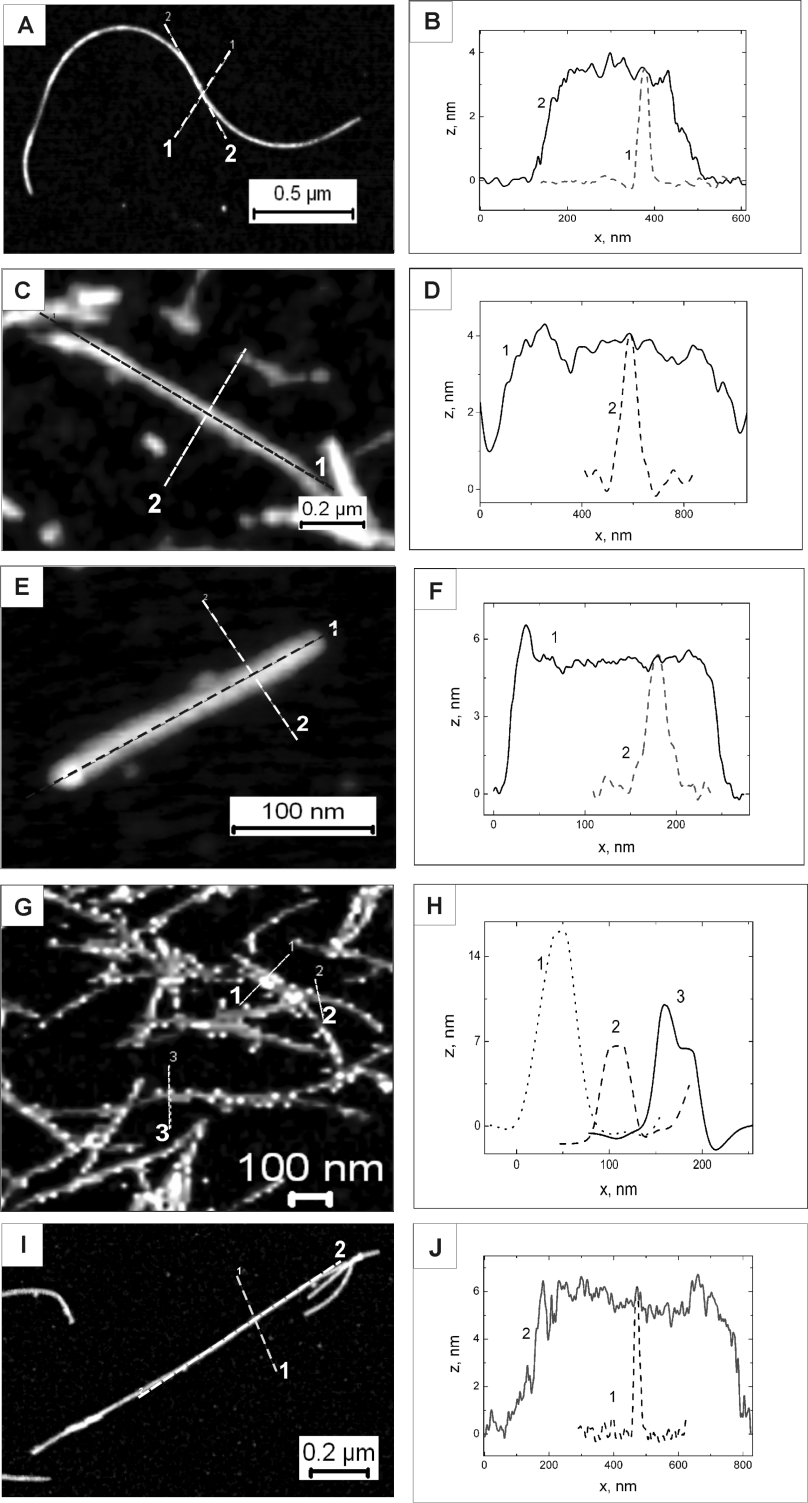
AFM topography images with cross-sectional and axial profiles. α-SynC141 fibril (A and B), fibrils reduced with tris(2-carboxyethyl)phosphine (TCEP) (C and D), biotinylated α-SynC141 fibrils after reduction with TCEP (E and F), biotinylated α-SynC141 fibrils after incubation with neutravidin-conjugated gold nanoparticles (G and H), and α-Syn fibrils (I and J). The dashed lines in the AFM images indicate the location where the cross-sections and axial measurements were taken.

According to the literature, the rapid fibril formation is usually initiated at low pH [12]. However, SDS-PAGE analysis showed the degradation of the α-SynC141 protein at pH 3.0. Therefore, to avoid degradation of α-SynC141, the fibrillization at a higher pH was performed. After five days of incubation, the fibrillar structures were evaluated by ThT fluorescence assay. AFM analysis revealed that α-SynC141 formed long, filamentous fibrils, with an average height of 3.12 ± 0.55 nm (Figure 2A,B) that was lower compared with that of wild type α-Syn protein fibrils (6.03 ± 1.17 nm, Figure 2I,J).

### Modification of α-SynC141 fibrils

Prior to the biotinylation of fibrils, 1 mM of tris(2-carboxyethyl)phosphine (TCEP) was added to generate free sulfhydryls and to prevent the formation of disulfide bonds between adjacent α-SynC141 molecules. The ThT fluorescence assay and AFM analysis confirmed that the reduction with TCEP did not disturb the fibrillar structures, and the average height of such fibrils was 3.84 ± 0.39 nm (Figure 2C,D). Sulfhydryl-reactive, biotin maleimide was used to modify the free –SH groups. Biotinylation was performed in PBS buffer (pH 7.5) and a 5-fold molar excess of biotin reagent was added to the mixture. To remove the excess of biotin, the α-SynC141 fibril sample was dialyzed.

The concentration of thiol groups in the samples, which depends on the modification step, was monitored using TNB reagent (Table 1). Less than 1% of free thiol groups were detected both in the initial solution of α-SynC141 and after the fibrillization. Notwithstanding that, the main fraction of the initial solution of α-SynC141 contained dimers, which could be reduced to monomers by TCEP treatment (a gel-filtration analysis, data not shown), and the formation of fibrils was similar to that of the wild type α-Syn. After the reduction of α-SynC141 fibrils with TCEP and subsequent dialysis, the concentration of free sulfhydryl groups was similar to the initial concentration of α-SynC141 (before the fibril formation), but the number of thiol groups decreased by 100-fold after the biotinylation step (Table 1).

**Table 1:** Detection of sulfhydryl groups during modification of α-SynC141 fibrils. The initial concentration of α-SynC141 was 4 × 10^−3^ M.

synthesis step	concentration of –SH groups [M]

before fibrillization	1.67 ± 0.76 × 10^−5^
after fibrillization	3.55 ± 1.56 × 10^−5^
after fibrillization and reduction with TCEP	4.00 ± 0.50 × 10^−3^
after fibrillization, reduction, biotinylation	5.94 ± 1.19 × 10^−5^

The AFM analysis showed that after labelling, the fibrils remained long and unbranched, yet became thicker than non-biotinylated fibrils, and were of 5.27 ± 1.02 nm height (Figure 2E,F). The modification of the α-SynC141 protein with biotin before the fibrillization was also performed, but no fibril structures were observed after such alteration (data not shown).

To construct multicomponent nanostructures, biotinylated α-SynC141 fibrils were incubated with neutravidin-conjugated gold nanoparticles for 1 to 5 days. The spherical gold nanoparticles (10 nm) had two covalently bound neutravidin molecules that could be coupled to two fibrils through interaction with biotins. After incubation at 4 °C with neutravidin-conjugated gold nanoparticles, the samples were analyzed by AFM. Figure 2G,H demonstrates the AFM topography image obtained and the profile of biotinylated α-SynC141 fibrils obtained after incubation with neutravidin-conjugated gold nanoparticles, respectively. Hybrid nanostructures were observed in the sample containing fibrils and neutravidin-conjugated gold nanoparticles. These nanostructures were composed of long fibrils with attached homogeneous derivatives whose size (≈10 nm) corresponded to that of the gold nanoparticles. The cross sections in different locations confirmed the height of biotinylated α-SynC141 fibrils (≈5.5 nm), and the height of fibrils with attached neutravidin-conjugated gold nanoparticles (≈10–14 nm). The mean height of modified fibrils with neutravidin-conjugated gold nanoparticles, measured from the image, was 7.60 ± 2.44 nm.

**Figure 3:**
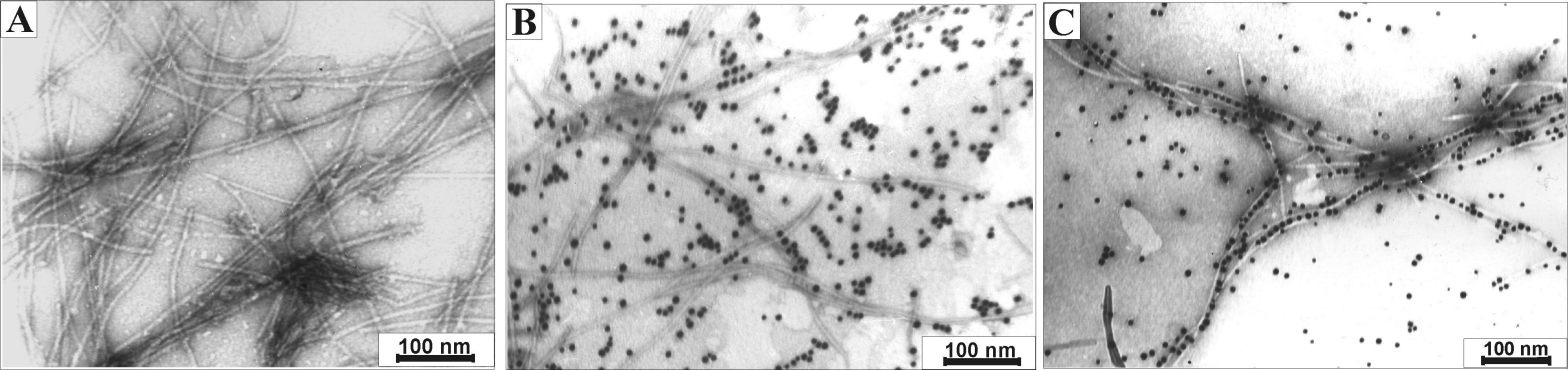
TEM image of modified α-SynC141 fibrils. α-SynC141 fibrils modified with biotin (A). α-SynC141 fibrils modified with biotin and neutravidin-conjugated gold nanoparticles after 24 hours of incubation (B). Modified α-SynC141 fibrils with biotin and neutravidin-conjugated gold nanoparticles after a week of incubation (C). The scale bar indicates 100 nm. The magnification was 100,000.

The same samples were analyzed by TEM, which confirmed the attachment of gold nanoparticles to fibrils formed by α-SynC141 (Figure 3). The assembly process was found to be time dependent. After 24 hours of incubation, neutravidin-conjugated nanoparticles were found evenly scattered among the biotinylated fibrils. A number of fibrils involved in the nanoladder formation were evaluated by analyzing randomly chosen areas in the TEM images. It was determined that approximately 5% of fibrils formed nanoladders after 24 hours of incubation. However, a higher degree of aggregation was observed after 5 days of incubation at 4 °C (Figure 3C). The fraction of the hybrid, ladder-like nanostructures increased up to ≈30% (Figure 3A,B). Neutravidin-conjugated gold nanoparticles were selectively attached to biotinylated fibrils through a neutravidin–biotin interaction. Although this interaction is extremely strong, the duration of assembly may presumably depend upon different factors including temperature, pH, and ionic strength. An incubation temperature of 4 °C was chosen to preserve the neutravidin-conjugated nanoparticles.

## Discussion

Self-assembling biomolecules such as DNA, peptides or proteins are of special interest in the design and construction of nanoscale materials and nanostructures. Proteins provide masterful examples of complex self-assembling nanostructures with properties and functionalities beyond the reach of any human-made materials [44]. The fibrils formed by amyloid proteins or peptides are nanostructures with unique physical and chemical stability [45], and therefore are favourable for the construction of complex nanostructures.

In this work, we chose the 140-amino acid protein, α-Syn, which is a very useful building block in nanobiotechnology since the purification of α-Syn is relatively simple, and a high yield of this protein can be achieved [46]. α-Syn has an unfolded C-terminal region and the NAC region (amino acids 61–95) that is responsible for aggregation. It was also demonstrated that the C-terminal is very important in the oligomerization process [47] and has a chaperone-like activity [11]. Therefore, a α-SynC141 mutant with one additional amino acid (cysteine) at the *C*-terminus was constructed to obtain a modifiable protein with a propensity to form fibrils. We showed that the α-SynC141 mutant retained the properties of a wild type protein, and, under the same conditions, both the yield and the purity of the mutant protein were similar to those of α-Syn. The theoretical molecular mass of α-SynC141 (14.46 kDa) was confirmed by mass spectrometry. However, a band corresponding to about 18 kDa was observed in SDS-PAGE (Figure 1). Assuming that a very acidic *C*-terminus of α-Syn weakly interacts with SDS, the electrophoretic migration of α-SynC141 to a position corresponding to 18 kDa seems plausible [48]. An additional ≈70 kDa band appeared after the two-step purification of α-SynC141. This band may indicate the formation of α-SynC141 tetramers. The formation of α-Syn tetramers was observed when α-Syn was isolated from neuronal and non-neuronal cell lines, brain tissue and living human cells, and analyzed under non-denaturing conditions [49].

The purified protein was used to study the modification and fibrillization of α-SynC141. According to the literature, one of the main factors which induces fibrillization is low pH [50]. However, at low pH values, the attempts to form fibrils from the intact protein were unsuccessful, since the recombinant protein was found to degrade into smaller fragments during the fibrillization procedure. For this reason, and to avoid degradation of the protein, α-SynC141 was fibrillized at pH 7.5 [51]. After 5 days of incubation, long filaments were observed in the fibril sample. The height of α-SynC141 fibrils was 3.12 ± 0.55 nm. Khurana et al. proposed a model for the hierarchical assembly of α-synuclein into amyloid fibrils when the pairs of protofilaments wind together to form protofibrils, and each of two protofibrils wind to form a fibril [52]. In this work, the observed fibrillar structures of α-SynC141 can be referred to as protofilaments due to their similarity with α-Syn protofilaments (3.8 ± 0.6 nm) previously described [52]. In contrast, the height of the fibrils formed during the control experiments of α-Syn aggregation was around 6 nm, which corresponded to the height of the wild type α-Syn fibrils (6–12 nm) observed by other researchers [20,53]. It was previously demonstrated that the protofilaments were stabile only in the presence of heparin molecules, otherwise protofilaments intertwined into higher level organized structures. It was proposed that charges on synuclein molecules bind heparin molecules and become stabilized as protofilaments, whereas in the absence of heparin, the protofilaments are highly charged and quickly intertwine with other protofilaments to yield stable protofibrils [52]. In our case, it might be speculated that stabilization of α-SynC141 protofilaments against further intertwining was due to the formation of S–S bonds between α-SynC141 monomers. It might also be proposed that only one part of the α-SynC141 dimer participated in a fibrillization process, whereas another part showed a shielding effect against further formation of protofibrils or/and fibrils. The oxidation of sulfhydryl groups to form disulfide bonds was confirmed by quantifying the free sulfhydryl groups in the α-SynC141 fibril sample. Therefore, the reduction of S–S bonds was necessary to obtain free thiol groups. After reduction with TCEP, the fibril sample was analyzed using ThT and AFM, and it was confirmed that TCEP did not disturb the fibrillar structures. Subsequently, these fibrils with exposed –SH groups were biotinylated. The formation of thicker fibrils as well as the absence of free sulfhydryls verified the successful biotinylation (Figure 2E,F). These results substantiate the findings that amyloid fibrils, including α-Syn fibrils, have highly stable mechanical properties that are essential for the functionalization.

**Figure 4:**
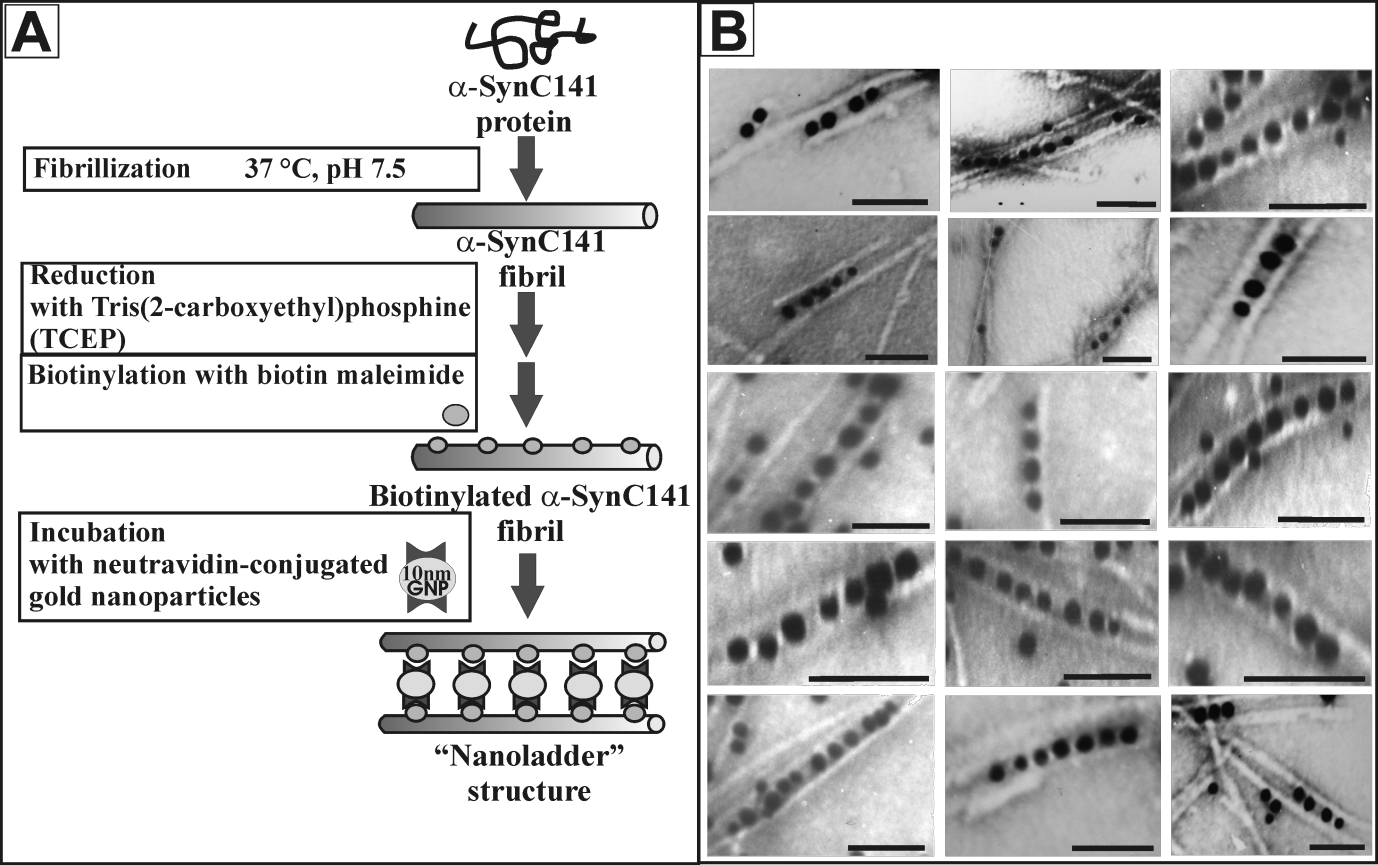
Schematic illustration of the formation of the hybrid nanostructure (nanoladder) (A). TEM images of the ladder-like nanostructures composed of two biotinylated α-SynC141 fibrils coupled with neutravidin-conjugated gold nanoparticles (B). The scale bars are 50 nm.

After the attachment of neutravidin-conjugated gold nanoparticles, heteromorphous nanostructures were observed. These nanostructures (Figure 4B) confirmed the theoretical scheme of formation of hybrid nanostructures (Figure 4A), where gold nanoparticles linked two fibrils through the biotin–neutravidin interaction. Neutravidin-conjugated gold nanoparticles can attach to one fibril, when two neutravidin molecules that are present on the surface of gold nanoparticle bind to biotin moieties on the same fibril. The application of the biotin–streptavidin (avidin) interaction, which is known to be one of the strongest interactions in nature, offers a broad range of possibilities with respect to functionalization of fibrils, since many streptavidin-conjugated components (enzymes and metal nanoparticles) are already available.

Depending on the final aim, two strategies for functionalization may be used since the modifications can be performed before or after fibrillation. The preassembly modification is usually chosen when it is essential to monitor the oligomerization process in vivo as well as in vitro. Various fluorescent labels have been used for a preassembly modification, namely, a thiol-reactive maleimide probe based on 2-(2-furyl)-3-hydroxychromone [37], fluorogenic biarsenical compounds [38], or a yellow fluorescent protein variant, Venus [39]. The main challenge using preassembly modification is to maintain the aggregation tendency of the target protein. Therefore, the postassembly modification is more advantageous in constructing the nanostructures. Due to extremely stable physical and chemical properties, amyloid fibrils can be successfully modified using a variety of modification agents. The fibrils of α-Syn and other proteins or peptides were successfully utilized as biotemplates for the enzyme immobilization [25–26] or for the production of nanowires [30,45,54] and the construction of biosensing platforms [22].

Herein we present a feasible method to perform the postassembly bio-functionalization of amyloid fibrils. Our results demonstrate the robustness and stability of such amyloid. The resultant multicomponent nanostructure (nanoladders) should contribute to a better understanding of regularities of self-assembly, and has the potential to be exploited for the development of novel functionalized nanomaterials or nanodevices.

## Conclusion

Fibrous nanostructures offer new opportunities for the development of the next generation of micro- and nano-devices with possible industrial applications. The α-SynC141 protein is an excellent building block for the construction of nanostructured hybrid materials. In this work we demonstrated that: (1) the recombinant α-SynC141 protein, which contains an additional cysteine residue introduced at position 141, formed filamentous fibrils similar to those observed for α-Syn, and (2) the α-SynC141 fibrils could be modified with biotin and, in the presence of neutravidin-conjugated gold nanoparticles, subsequently assembled into the ladder-like structures.

## Experimental

### Construction of α-syn mutant (α-synC141)

The expression plasmid (pRK172) harbouring gene for human α-Syn was kindly provided by Dr. L. A. Morozova-Roche and Dr. M. Malisauskas. Wild-type α-Syn does not contain cysteine residues. The single cysteine mutant (α-SynC141) was constructed using primers T7Pro (5′-TAATACGACTCACTATAGCG-3′) and SyncysR (5′-TACTCGAGTTAACAGGCTTCAGGTTCGTAG-3′ (Metagene). The obtained PCR fragment was digested with *Xba*I and *Xho*I restriction endonucleases (Thermo Scientific) and inserted into the corresponding sites of the pET21a(+) vector (Novagen). The ligation mixture was transferred into *E*. *coli* DH5α. Positive clones were identified by PCR screening and restriction analysis. The presence of the desired mutation was verified by DNA sequencing (Sequencing Center, Institute of Biotechnlogy, Vilnius University).

### Expression and purification of mutant α-SynC141

Wild-type α-Syn and α-SynC141 mutant were purified as described by Der-Sarkissian et al. [46], with some modifications. For the preparation of recombinant proteins, pET21α-SynC141 and pRK172 plasmids containing α-*synC*141 and α-*syn* genes respectively were introduced into *E. coli* BL21 (DE3) cells by electroporation. 5 mL of the overnight culture were used to inoculate 1 L of NB media containing ampicillin (50 μg/mL). The cells were grown at 37 °C until the OD_600_ reached 0.8; the protein expression was induced by the addition of 0.2 mM IPTG, and cell growth continued at 30 °C for 18 h. The biomass was collected by centrifugation at 4000*g* and resuspended in 50 mM Tris-HCl buffer (pH 8.0), containing 0.1 mM EDTA, 0.2 mM PMSF, and 500 mM NaCl. The cell lysate was heated at 100 °C for 10 min and subsequently centrifuged at 13200*g* for 20 min. The supernatant was dialyzed against 50 mM Tris-HCl, pH 8.0, 1 mM dithiothreitol, 1 mM EDTA, pH 8.0, and loaded into a HiTrap ANX column (GE Healthcare) equilibrated in the same buffer. Proteins were eluted using a linear gradient of 0–1 M NaCl. Fractions containing α-SynC141 were pooled and additionally applied to a HiTrap Q XL column (GE Healthcare) and were identified by 15% SDS-PAGE. The α-Syn protein, expressed and purified in the same manner as α-SynC141, was used for control assays. Protein purity was evaluated by gel electrophoresis. Mass spectrometry verified the presence of the cysteine residue in the *C* terminal.

### α-SynC141 and α-Syn fibril formation

Lyophilized proteins were dissolved in the buffer: 50 mM Tris-HCl, 100 mM NaCl, pH 7.5 to a final concentration (360 μM or 5 mg/mL) and were fibrillized at 37 °C by continuous shaking for 5 days [51].

### Thioflavin T (ThT) assay

The fibril formation was evaluated by monitoring ThT (Fluka, Germany) fluorescence. The ThT solution, containing 5 μM of ThT in PBS (pH 7.4) buffer, was mixed with 5 μM of fibril solution (calculated from the initial concentration of protein prior to fibrillization) and incubated for 10 minutes at room temperature. Fluorescence emission spectra of ThT, excited at 450 nm, were recorded between 460 and 600 nm on a PerkinElmer LS55 luminescence spectrometer using excitation and emission bandwidths of 5 nm.

### Modification of fibrils

Prior to the labelling with biotin, the thiol groups of proteins were reduced in the presence of 1 mM of TCEP (AppliChem) and then the mixture was dialyzed against 50 mM sodium phosphate buffer (pH 6.5). For the preparation of 10 mM reaction solution, 4.5 mg of biotin maleimide (Sigma-Aldrich) was initially dissolved in DMSO (Sigma-Aldrich) to achieve 50 mM concentration, and then diluted with 50 mM sodium phosphate buffer (pH 6.5) to the final concentration 20% of DMSO. For the biotinylation, a 10 mM biotin maleimide reaction solution was added to achieve a 5-fold molar excess of biotin over the protein. After 2 hours of biotinylation at room temperature, the mixture was dialyzed against an appropriate buffer.

To evaluate the efficiency of biotinylation, free sulfhydryl groups were analyzed with Ellman’s reagent (5,5'-dithiobis-(2-nitrobenzoic acid)) (Thermo Scientific): 4 mg of Ellman’s reagent was dissolved in 1 mL of reaction buffer (0.1 M sodium phosphate, 1 mM EDTA, pH 8.0) to obtain the 10 mM concentration. The fibril samples were tested by preparing a tube containing 18 μL of Ellman’s reagent solution, 90 μL of fibril solution and reaction buffer to 1 mL. The mixture was incubated at room temperature for 15 min. The absorbance was recorded by a spectrophotometer at 412 nm. The concentration of free sulfhydryl in the sample was calculated using a molar extinction coefficient of 14,150 M^−1^cm^−1^ for 2-nitro-5-thiobenzoic acid (TNB).

Biotinylated fibrils were incubated with neutravidin-conjugated 10 nm gold nanoparticles (Nanopartz, USA). Assuming that one neutravidin-conjugated gold nanoparticle can interact with 8 biotins, an appropriate volume of a nanoparticle suspension was added to the biotinylated fibril mixture to achieve an 8-fold molar excess of fibrils over nanoparticles. Mixtures of fibrils and nanoparticles were incubated for 1 to 5 days at 4 °C.

### Atomic force microscopy

The presence of fibrils was confirmed by atomic force microscopy (AFM). A small amount (typically 1 μL) of fibril solution was spread on a mica surface. The immobilization was allowed to proceed for 5 to 20 min, at 23 °C at a relative humidity of about 45%. The density of fibrillar structures on mica was increased or decreased by the immobilization time or the dilution rate. After adsorption, the surfaces were rinsed with distilled water, dried and visualized using the standard contact and tapping AFM modes by scanning probe microscope SPM D3100/Nanoscope IVa (Veeco, now Bruker). Two types of silicon tips, OTESPA and SNL (Bruker), were used. The images were processed by the Scanning Probe Image Processor, Version 5.1.0 software (Image Metrology, Denmark). The standard deviation was calculated according the formula:


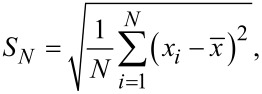


where *N* is number of positions, *x_i_* is the height at the *i* position, and 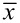 is the mean value of all heights.

### Transmission electron microscopy (TEM)

1 μL of fibril solution was mounted on a carbon-coated palladium grid (400 mesh). The sample was dried at room temperature for 5–10 min, then negatively stained with 2% aqueous uranyl acetate solution (Reachim) and dried with filter paper. The sample grids were analyzed by a TEM (JEOL-JEM-100S, Japan) at an instrumental magnification of 25,000. The dimensions of the fibrils were obtained directly from the micrographs.
